# Supplementary figures and images for: Route of Application and Dose Evaluation of Dental Pulp Stem Cells for the Treatment of Sialadenitis Caused by Sjögren’s Syndrome: A Preclinical Study
Source: Biomedicines. 2025 Apr 28;13(5):1068. doi: 10.3390/biomedicines13051068 (PMC12109166; doi:10.3390/biomedicines13051068)

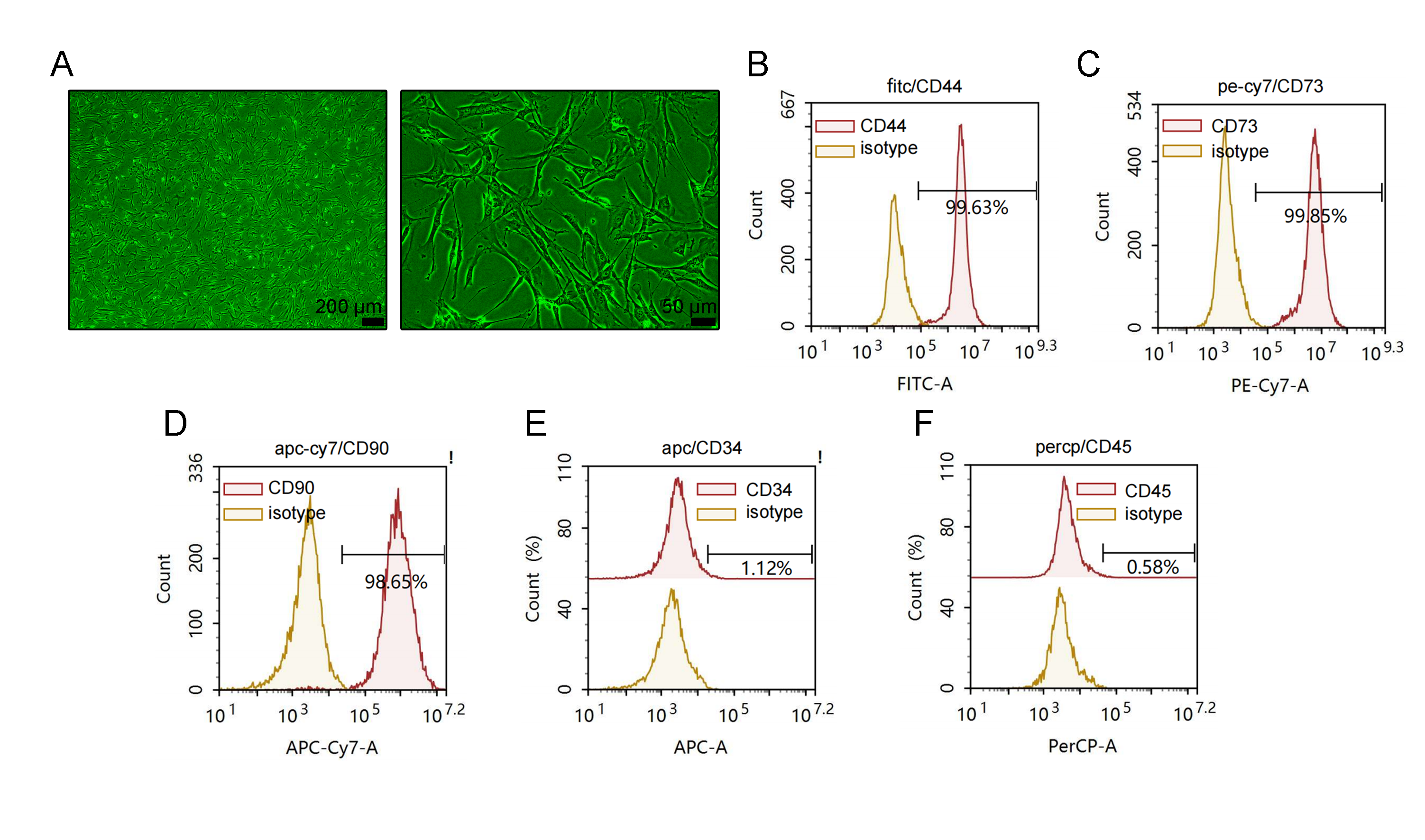

Supplement: Supplementary file 1 [file biomedicines-13-01068-s001.zip › biomedicines-3580122-figure s1.tif]
